# Comparison of the blood pressure management between sodium-glucose cotransporter 2 inhibitors and glucagon-like peptide 1 receptor agonists

**DOI:** 10.1038/s41598-022-20313-5

**Published:** 2022-09-27

**Authors:** Kazuo Kobayashi, Masao Toyoda, Nobuo Hatori, Hiroyuki Sakai, Takayuki Furuki, Kazuyoshi Sato, Yasuo Terauchi, Kouichi Tamura, Akira Kanamori

**Affiliations:** 1 Committee of Hypertension and Kidney Disease, Kanagawa Physicians Association, 3-1 Fujimicho naka-ku, Yokohama City, Kanagawa Prefecture Japan; 2 Department of Medical Science and Cardiorenal Medicine, Yokohama City University Graduate School of Medicine, Yokohama, Japan; 3 Division of Nephrology, Endocrinology and Metabolism, Department of Internal Medicine, Tokai University School of Medicine, lsehara, Japan; 4 Department of Endocrinology and Metabolism, Yokohama City University Graduate School of Medicine, Yokohama, Japan

**Keywords:** Endocrinology, Nephrology

## Abstract

The cardiovascular and renal protective effects of sodium-glucose cotransporter 2 inhibitors (SGLT-2is) and glucagon-like peptide 1 receptor agonists (GLP-1Ras) are enhanced by low/controlled blood pressure (BP). However, the BP-lowering efficacy of SGLT-2is and GLP-1Ras have not been compared directly. We compared the rates of achieving target BP with SGLT-2i and GLP-1Ra treatments in Japanese patients with type 2 diabetes mellitus (T2DM). This retrospective study included 384 SGLT-2i- and 160 GLP-1Ra-treated patients with BP > 130/80 mmHg before treatment. Inverse probability weighting methods using propensity scores were used in this study. The integrated odds ratios (OR) for BP control rates were calculated and clinical changes were analyzed using a generalized linear model. SGLT-2i treatment resulted in significantly higher BP control rates than that in the GLP-1Ra treatment (integrated OR = 2.09 [1.80, 2.43]). Compared with GLP-1Ra, SGLT-2i treatment demonstrated significantly larger decreases in diastolic BP, mean arterial pressure, and body weight (− 3.8 mmHg, *P* = 0.006; − 4.1 mmHg, *P* = 0.01; and − 1.5 kg, *P* = 0.008, respectively) and increased annual estimated glomerular filtration rate (eGFR; 1.5 mL/min/1.73 m^2^/year, *P* = 0.04). In T2DM patients with poorly controlled BP, compared with GLP-1Ra, SGLT-2i treatment significantly improved BP management and increased eGFR.

## Introduction

As rosiglitazone treatment is associated with increased cardiovascular risk, especially heart failure and myocardial infarction^1^, the United States Food and Drug Administration (FDA) requires non-inferiority randomized placebo-controlled trials, known as cardiovascular outcome trials (CVOT), to assess the cardiovascular risk of new types of drugs used for treating type 2 diabetes mellitus (T2DM)^2^. Compared to placebo, treatment with dipeptidyl peptidase-4 inhibitors (DPP4is) demonstrated non-inferiority for major adverse cardiac outcomes^3–6^. However, treatment with saxagliptin was associated with an increased risk of heart failure^5^. In contrast, several CVOTs have demonstrated the predominance of sodium-glucose cotransporter-2 inhibitors (SGLT-2is)^7–9^ and glucagon-like peptide-1 receptor agonists (GLP-1Ras) in preventing major cardiovascular events compared to placebo^10–13^. Furthermore, in several CVOTs, SGLT-2i and GLP-1Ra treatments have been associated with superior renal outcomes^7–9,14^. Based on these findings, which support the cardiovascular and renal protective effects of SGLT-2i and GLP-1Ra, these two types of antidiabetics are highly recommended in guidelines^15,16^. The mechanisms underlying the cardiovascular and renal protective effects are not completely understood. It seems that not only their hypoglycemic effect but also other effects, such as lowering blood pressure (BP) and body weight (BW), and improving insulin resistance or lipid profile, are involved therein. The hypoglycemic effects of SGLT-2i are not essential for its protective effects. A DAPA-HF study investigating dapagliflozin^17^ and EMPEROR-reduced clinical trials investigating empagliflozin^18^ demonstrated their predominance in cardiovascular outcomes, whereas a DAPA-CKD trial showed their predominance in renal outcomes in patients with or without diabetes compared to placebo^19^.

We previously performed a retrospective observational survey of 624 T2DM patients with chronic kidney disease (CKD) and demonstrated that SGLT-2i treatment decreased the urine albumin-to-creatinine ratio (ACR) in clinical practice^20^. We observed that the BP-lowering effect correlated with the renoprotective effect of SGLT-2i^21–24^. We also performed a retrospective observational survey that included 547 GLP-1Ra-treated T2DM patients (data not published). Using these two reports, we directly compared the renal and cardioprotective effects of SGLT-2i and GLP-1Ra by propensity score (PS) matching^25^. This analysis demonstrated the significant predominance of SGLT-2i over GLP-1Ra on the renal composite outcome in T2DM patients, and that, in SGLT-2i-treated patients, the decrease in BP was significantly correlated with the decrease in ACR (*P* = 0.04)^25^.

In addition to hypoglycemic effects, SGLT-2is and GLP-1Ras showed multifaceted effects, such as a decrease in BP and BW, and improvement in lipid profiles. These multifaceted effects may influence the organ-protective outcomes (cardiovascular and renal outcomes) of SGLT-2is and GLP-1Ras. However, the mechanism underlying these outcomes are not completely understood. To date, our retrospective observational study showed the predominance of SGLT-2is over GLP-1Ras in the renal outcome, and the importance of the decrease in BP for the improvement of renal outcome by SGLT-2is. However, our study included both BP-controlled and BP-uncontrolled patients at baseline. For example, the DAPA-CKD study showed renoprotective effects in patients regardless of baseline BP^19^. BP-controlled patients at baseline may be managed to prevent excessive hypotension during SGLT-2i treatment. Therefore, the analysis including BP-controlled patients at baseline in the retrospective study cannot elucidate the role of the decrease in BP in renoprotective effects. For these reasons, we hypothesized that SGLT-2is are predominance for BP management in patients with hypertension and that they demonstrate better renal outcomes compared to GLP-1Ras. Furthermore, to the best of our knowledge, no study has reported the antihypertensive effect of SGLT-2is compared to GLP-1Ras, exclusively in BP-uncontrolled patients with hypertension. In the future, our study may support preferential treatment with SGLT-2is in patients with hypertension and DM.

Furthermore, our previous study included both BP-controlled and BP-uncontrolled patients. We believe that the analysis of patients with uncontrolled BP at baseline can better demonstrate the causal relationship between the decrease in BP and renal effects.

Using clinical databases, we posited that appropriate BP control is an important mechanism underlying the renoprotective effects of SGLT-2is and GLP-1Ras. However, a direct comparison of the BP-lowering effects of these two types of antidiabetic drugs has not been reported. Therefore, this study aimed to compare differences in BP control rates induced by SGLT-2i and GLP-1Ra treatments in Japanese patients with T2DM.

## Results

### Clinical characteristics of SGLT-2i- and GLP-1Ra-treated patients at baseline

Supplementary Fig. S1 shows a schematic of the subject selection procedure. We included 384 SGLT-2i- and 160 GLP-1Ra-treated patients in the comparative analysis. The median duration of treatment was 32 months (range 12–55 months) for the SGLT-2i group and 48.5 months (range 12–123 months) for the GLP-1Ra group. Table 1 presents the clinical characteristics of the SGLT-2i- and GLP-1Ra-treated patients at baseline. No significant differences were observed between the groups in terms of values of systolic BP (SBP) and ACR, logarithmic value of ACR (LnACR), or concomitant use of pioglitazone, calcium channel blockers, and statins. However, significant differences were observed between the groups with respect to other clinical characteristics, including age, sex, BW, body mass index (BMI), diastolic BP (DBP), mean arterial pressure (MAP), glycated hemoglobin A_1c_ (HbA_1c_), estimated glomerular filtration rate (eGFR), duration of treatment, and the use of sulfonylurea metformin, insulin, renin-angiotensin system inhibitors (RASis), β-blockers, and diuretics (loop and thiazides) (*P* < 0.001, = 0.003, < 0.001, = 0.003, = 0.003, = 0.01, < 0.001, < 0.001, < 0.001, = 0.01, < 0.001, < 0.001, = 0.04, = 0.02, and < 0.001, respectively).

**Table 1:** The clinical characteristics of SGLT2i-treated and GLP1Ra-treated patients at baseline.

	SGLT2i-treated patients (n = 384)	GLP1Ra-treated patients (n = 160)	P-value
Age (year-old)	57.9 ± 11.2	63.5 ± 13.5	< 0.001^b^
Sex (female (%))	126 (32.8%)	74 (46.3%)	0.003^a^
BW (kg)	80.1 ± 16.2	73.4 ± 17.7	< 0.001^a^
BMI	29.3 ± 4.9	28.0 ± 5.4	0.003^a^
SBP (mmHg)	141.1 ± 12.8	140.9 ± 11.9	0.44^a^
DBP (mmHg)	81.6 ± 11.2	78.6 ± 11.2	0.003^a^
MAP (mmHg)	101.4 ± 9.5	99.4 ± 8.9	0.01^a^
HbA_1c_ (mmol/mol (%))	62.5 ± 13.5 (7.9 ± 1.2)	68.6 ± 17.9 (8.4 ± 1.6)	< 0.001^a^
eGFR (mL/min/1.73 m^2^)	79.4 ± 21.4	66.7 ± 25.2	< 0.001^a^
ACR (mg/gCr)	38.4 [13.0, 125.5]	28.2 [13.5, 142.5]	0.88^c^
LnACR	1.62 ± 0.66	1.66 ± 0.78	0.27^a^
Duration of the treatment (month)	31.9 ± 10.7	55.2 ± 31.4	< 0.001^a^
**The concomitant treatment**			
SU	114 (29.7%)	30 (18.8%)	0.01^b^
Metformin	231 (60.2%)	61 (38.1%)	< 0.001^b^
Insulin	87 (22.7%)	72 (45.0%)	< 0.001^b^
Pioglitazone	75 (19.5%)	23 (14.4%)	0.15^b^
RAS inhibitor	213 (55.5%)	104 (65.0%)	0.04^b^
CCB	189 (49.2%)	91 (56.9%)	0.10^b^
βblocker	46 (12.0%)	31 (19.4%)	0.02^b^
Diuretics (loops and thiazides)	25 (6.5%)	26 (16.3%	< 0.001
Statin	234 (60.9%)	97 (60.6%)	0.95^b^

Values represent mean ± standard difference, n (n/total %), or medium [25% quantile, 75% quantile].

*BMI* body mass index, *BW* body weight, *CCB* calcium channel blocker, *DBP* diastolic blood pressure, *eGFR* estimated glomerular filtration rate, *GLP1Ra* glucagon-like-1 receptor agonist, *HbA*_*1c*_ glycated hemoglobin A_1c_, *LnACR* logarithmic value of urine albumin-to-creatinine ratio, *MAP* mean arterial pressure, *RAS* renin aldosterone system, *SBP* systolic blood pressure, *SGLT2i* sodium glucose cotransporter inhibitor, *SU* sulfonyl urea.

^a^Chi square test.

^b^Unpaired t test.

^c^Mann–Whitney rank-sum test.

### Cohort models using inverse probability weighting estimation

The clinical characteristics of SGLT-2i- and GLP-1Ra-treated patients after inverse probability weighting (IPW) are shown in Tables 2, 3, and 4, where the weighting methods utilized were the average treatment effect (ATE), average treatment effect on the treated (ATT), and stabilized ATE, respectively. Furthermore, two different methods were used for the adjustment of IPW; “weight truncation” that consisted of the exclusion of weights larger than 99 percentiles (model A), or “weight trimming” that consisted of excluding extreme PS values and including only the patients with PS ranging from 0.05 to 0.95 (model B), who were selected for further analysis. The distribution of PS in each group is shown in Supplementary Fig. S3. The C-index of the calculated PS is 0.90.

**Table 2:** The clinical characteristics of SGLT2i-treated and GLP1Ra-treated patients after ATE weighting.

	Model A (the truncation on 99 percentiles)			Model B (the trimming by 0.05 ≦ PS ≦ 0.95)		
	SGLT2i (n = 501^a^)	GLP1Ra (n = 513^a^)	Standardized difference	SGLT2i (n = 338^a^)	GLP1Ra (n = 309^a^)	Standardized difference
Age (year-old)	59.7 ± 11.5	63.2 ± 12.4	0.29	62.8 ± 11.0	64.8 ± 12.4	0.17
Sex (female)	34.1 (%)	41.9 (%)	0.16	38.9 (%)	45.0(%)	0.13
BW (kg)	78.2 ± 15.9	72.7 ± 15.5	0.35	74.3 ± 14.4	71.9 ± 18.0	0.14
BMI	28.8 ± 4.8	27.5 ± 4.8	0.29	27.9 ± 4.3	27.6 ± 5.6	0.06
SBP (mmHg)	141.1 ± 13.1	142.9 ± 13.8	0.14	140.9 ± 13.5	141.1 ± 12.0	0.01
DBP (mmHg)	81.0 ± 11.3	79.0 ± 11.8	0.17	79.2 ± 11.4	76.9 ± 11.5	0.20
MAP (mmHg)	101.0 ± 9.5	100.3 ± 9.8	0.08	99.8 ± 9.6	98.3 ± 8.9	0.16
HbA_1c_ (mmol/mol (%))	64.4 ± 14.8 (8.0 ± 1.4)	64.7 ± 15.3 (8.1 ± 1.4)	0.02	66.5 ± 16.0 (8.2 ± 1.5)	65.8 ± 16.3 (8.2 ± 1.5)	0.04
eGFR (mL/min/1.73m^2^)	76.4 ± 21.6	72.9 ± 22.0	0.16	71.0 ± 19.9	69.3 ± 22.0	0.08
LnACR	1.66 ± 0.67	1.62 ± 0.68	0.06	1.67 ± 0.71	1.65 ± 0.82	0.03
Duration of the treatment (month)	33.4 ± 10.7	35.3 ± 25.7	0.10	36.4 ± 9.6	37.9 ± 21.9	0.09
**The concomitant treatment**						
SU	25.5 (%)	25.2 (%)	0.01	18.4 (%)	21.4 (%)	0.08
Metformin	55.1 (%)	56.1 (%)	0.02	46.6 (%)	45.0 (%)	0.03
Insulin	29.7 (%)	34.1 (%)	0.09	40.2 (%)	41.4 (%)	0.02
Pioglitazone	17.6 (%)	9.4 (%)	0.24	15.1 (%)	13.6 (%)	0.04
RAS inhibitor	58.7 (%)	63.2 (%)	0.09	63.6 (%)	58.9 (%)	0.10
CCB	48.6 (%)	47.3 (%)	0.03	50.0 (%)	51.8 (%)	0.04
β blocker	16.1 (%)	15.0 (%)	0.03	20.8 (%)	22.1 (%)	0.03
Diuretics (loops and thiazides)	6.6 (%)	6.6 (%)	0.003	7.4 (%)	7.4 (%)	0.003
Statin	57.2 (%)	52.2 (%)	0.10	55.2 (%)	52.4 (%)	0.05

Values represent the mean ± standard difference, or n (n/total %).

*ATE* average treatment effect, *BMI* body mass index, *BW* body weight, *CCB* calcium channel blocker, *DBP* diastolic blood pressure, *eGFR* estimated glomerular filtration rate, *GLP1Ra* glucagon-like-1 receptor agonist, *HbA*_*1c*_ glycated hemoglobin A_1c_, *LnACR* logarithmic value of urine albumin-to-creatinine ratio, *MAP* mean arterial pressure, *PS* propensity score, *RAS* renin aldosterone system, *SBP* systolic blood pressure, *SGLT2i* sodium glucose cotransporter inhibitor, *SU* sulfonylurea.

^a^Calculated number of participants after weighting.

**Table 3:** The clinical characteristics of SGLT2i-treated and GLP1Ra-treated patients after ATT weighting.

	Model A (the truncation on 99 percentiles)			Model B (the trimming by 0.05 ≦ PS ≦ 0.95)		
	SGLT2i (n = 384^a^)	GLP1Ra (n = 353^a^)	Standardized difference	SGLT2i (n = 224^a^)	GLP1Ra (n = 193^a^)	Standardized difference
Age (year-old)	57.9 ± 11.2	63.0 ± 11.9	0.44	61.2 ± 10.8	65.4 ± 11.9	0.37
Sex (female)	32.8 (%)	39.9 (%)	0.15	38.8 (%)	45.6 (%)	0.14
BW (kg)	80.1 ± 16.2	72.4 ± 14.4	0.50	75.5 ± 14.9	70.9 ± 17.6	0.29
BMI	29.3 ± 4.9	27.2 ± 4.5	0.43	28.2 ± 4.5	27.4 ± 5.6	0.16
SBP (mmHg)	141.1 ± 12.8	143.9 ± 14.5	0.20	140.8 ± 13.1	141.3 ± 11.9	0.03
DBP (mmHg)	81.6 ± 11.2	79.2 ± 12.1	0.21	79.3 ± 11.5	76.4 ± 11.3	0.26
MAP (mmHg)	101.4 ± 9.5	100.7 ± 10.2	0.07	99.8 ± 9.7	98.0 ± 8.7	0.20
HbA_1c_ (mmol/mol (%))	62.5 ± 13.5 (7.9 ± 1.2)	92.9 ± 13.6 (7.9 ± 1.2)	0.03	64.2 ± 15.0 (8.0 ± 1.4)	63.9 ± 14.9 (8.0 ± 1.4)	0.02
eGFR (mL/min/1.73 m^2^)	79.4 ± 21.4	75.7 ± 19.9	0.18	73.4 ± 20.1	71.2 ± 19.9	0.11
LnACR	1.62 ± 0.66	1.60 ± 0.63	0.04	1.62 ± 0.71	1.64 ± 0.82	0.03
Duration of the treatment (month)	31.9 ± 10.7	26.2 ± 15.8	0.43	35.4 ± 9.7	33.0 ± 18.0	0.18
**The concomitant treatment**						
SU	29.7 (%)	28.1 (%)	0.04	22.3 (%)	23.8 (%)	0.04
Metformin	60.2 (%)	64.3 (%)	0.09	51.3 (%)	48.7 (%)	0.05
Insulin	22.7 (%)	29.2 (%)	0.15	33.0 (%)	39.4 (%)	0.13
Pioglitazone	19.5 (%)	7.1 (%)	0.37	17.4 (%)	13.0 (%)	0.12
RAS inhibitor	55.5 (%)	62.3 (%)	0.14	60.3 (%)	58.5 (%)	0.04
CCB	49.2 (%)	42.9 (%)	0.13	51.8 (%)	50.8 (%)	0.02
β blocker	12.0 (%)	13.0 (%)	0.03	16.1 (%)	23.4 (%)	0.18
Diuretics (loops and thiazides)	6.5 (%)	2.3 (%)	0.53	7.6 (%)	4.1 (%)	0.31
Statin	60.9 (%)	48.4 (%)	0.25	60.7 (%)	47.2 (%)	0.27

Values represent the mean ± standard difference, or n (n/total %).

^a^Calculated number of participants after weighting.

*ATT* average treatment effect on the treated, *BMI* body mass index, *BW* body weight, *CCB* calcium channel blocker, *DBP* diastolic blood pressure, *eGFR* estimated glomerular filtration rate, *GLP1Ra* glucagon-like-1 receptor agonist, *HbA*_*1c*_ glycated hemoglobin A_1c_, *LnACR* logarithmic value of urine albumin-to-creatinine ratio, *MAP* mean arterial pressure, *PS* propensity score, *RAS* renin aldosterone system, *SBP* systolic blood pressure, *SGLT2i* sodium glucose cotransporter inhibitor, *SU* sulfonylurea.

**Table 4:** The clinical characteristics of SGLT2i-treated and GLP1Ra-treated patients after stabilized ATE weighting.

	Model A (the truncation on 99 percentiles)			Model B (the trimming by 0.05 ≦ PS ≦ 0.95)		
	SGLT2i (n = 351*)	GLP1Ra (n = 150*)	Standardized difference	SGLT2i (n = 240*)	GLP1Ra (n = 89*)	Standardized difference
Age (year-old)	59.6 ± 11.6	63.1 ± 12.5	0.30	62.8 ± 11.0	64.8 ± 12.4	0.18
Sex (female)	34.8 (%)	42.0 (%)	0.15	38.9 (%)	44.9 (%)	0.13
BW (kg)	78.2 ± 16.0	72.7 ± 15.5	0.35	74.3 ± 14.4	71.9 ± 1.8	0.15
BMI	28.8 ± 4.8	27.5 ± 4.8	0.29	27.9 ± 4.3	27.6 ± 5.6	0.07
SBP (mmHg)	141.3 ± 13.2	142.9 ± 13.8	0.12	140.9 ± 13.5	141.1 ± 12.1	0.01
DBP (mmHg)	80.9 ± 11.3	79.0 ± 11.9	0.17	79.2 ± 11.4	76.9 ± 11.5	0.20
MAP (mmHg)	101.1 ± 9.6	100.3 ± 9.8	0.08	99.8 ± 9.6	98.3 ± 9.0	0.16
HbA_1c_ (mmol/mol (%))	64.1 ± 14.6 (8.0 ± 1.3)	64.7 ± 15.3 (8.1 ± 1.4)	0.04	66.5 ± 16.0 (8.2 ± 1.5)	65.8 ± 16.4 (8.2 ± 1.5)	0.05
eGFR (mL/min/1.73 m^2^)	76.7 ± 21.6	64.7 ± 5.3	0.17	71.0 ± 20.0	69.3 ± 22.1	0.08
LnACR	1.65 ± 0.67	1.62 ± 0.68	0.05	1.67 ± 0.71	1.65 ± 0.82	0.03
Duration of the treatment (month)	33.3 ± 10.7	35.1 ± 25.7	0.11	36.4 ± 9.6	37.9 ± 22.0	0.11
**The concomitant treatment**						
SU	25.9 (%)	25.3 (%)	0.01	18.4 (%)	21.3 (%)	0.08
Metformin	55.8 (%)	56.0 (%)	0.003	46.7 (%)	44.9 (%)	0.03
Insulin	28.8 (%)	34.0 (%)	0.11	40.2 (%)	41.6 (%)	0.03
Pioglitazone	17.7 (%)	9.3 (%)	0.25	15.1 (%)	13.5 (%)	0.04
RAS inhibitor	58.1 (%)	63.3 (%)	0.11	63.6 (%)	58.9 (%)	0.08
CCB	49.3 (%)	47.3 (%)	0.04	50.0 (%)	51.7 (%)	0.03
β blocker	14.8 (%)	14.7 (%)	0.004	20.8 (%)	22.2 (%)	0.04
Diuretics (loops and thiazides)	6.6 (%)	6.7 (%)	0.008	7.1 (%)	7.8 (%)	0.05
Statin	58.1 (%)	52.0 (%)	0.12	55.2 (%)	52.2 (%)	0.04

Values represent the mean ± standard difference, or n (n/total %).

Abbreviation is same in Table 2.

^a^Calculated number of participants after weighting.

Trimming using the PS value excluded 204 patients from the analysis (166 patients had PS values > 0.95 and 38 patients had values < 0.05). When using the other method, five weights were larger than 99 percentiles and were trimmed.

### Primary outcome assessment

**Figure 1:**
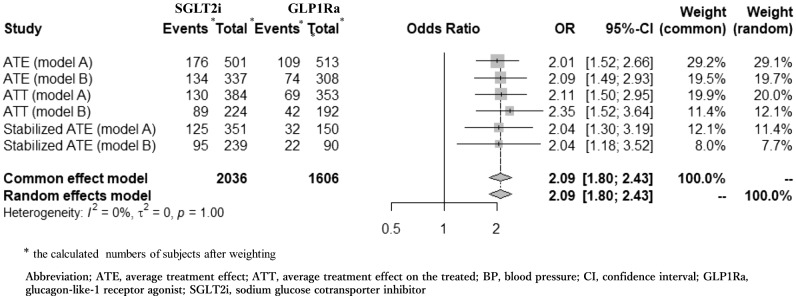
The integrated OR using six models by meta-analysis method. *Calculated number of participants after weighting. *ATE* average treatment effect, *ATT* average treatment effect on the treated, *BP* blood pressure, *CI* confidence interval, *GLP1Ra* glucagon-like-1 receptor agonist, *SGLT2i* sodium glucose cotransporter inhibitor.

Comparisons between the rates of achieving the target BP with SGLT-2i and GLP-1Ra treatments, based on the generalized linear model and integrated odds ratio (OR) analyses, are shown in Table 5 and Fig. 1, respectively. No significant differences were observed in primary outcomes between treatment groups when employing model A (P = 0.06, = 0.14, and 0.06 using ATE weighting, ATT weighting and stabilized ATE weighting, respectively). However, when using model B, the difference was statistically significant (*P* value was 0.03 using ATE weighting, 0.04 using ATT weighting, and 0.03 using stabilized ATE weighting). Figure 1 illustrates the integrated OR calculated using the meta-analysis method (2.09; 95% confidence interval [CI] 1.80–2.43). The weighted numbers of patients who reached the target BP after treatment (events) and the weighted numbers of all patients (total) using the six IPW models are shown on the left side of Fig. 1.

**Table 5:** The achievement ratio for BP control after SGLT2i treatment compare to GLP1Ra treatment by the analysis of the generalized linear model.

	OR* [95%CI]	P-value
ATE (model A)	2.01 [0.97, 4.20]	0.06
ATE (model B)	2.09 [1.08, 4.03]	0.03
ATT (model A)	2.11 [0.78, 5.73]	0.14
ATT (model B)	2.35 [1.03, 5.36]	0.04
Stabilized ATE (model A)	2.06 [0.98, 4.31]	0.06
Stabilized ATE (model B)	2.09 [1.08, 4.03]	0.03

The truncation on 99 percentiles is utilized in model A, and the trimming by 0.05 ≦ PS ≦ 0.95 is utilized in model B.

^a^Odds ratio for SGLT2i treatment compared to GLP1Ra treatment.

### Evaluation of standardized differences among the six utilized models

The standardized differences among the clinical baseline characteristics depending on the type of weighting model employed are shown in Supplementary Fig. S4. The median values and ranges of the standardized differences obtained when applying ATE weighting with truncation of values > 99 percentiles, ATE weighting with PS-based trimming (trimming by 0.05 ≦ PS ≦ 0.95), ATT weighting with truncation of values > 99 percentiles, ATT weighting with PS-based trimming, stabilized ATE weighting with truncation of values > 99 percentiles, and stabilized ATE weighting with PS-based trimming were 0.10 (0.003–0.35), 0.06 (0.003–0.20), 0.17 (0.03–0.53), 0.14 (0.02–0.37), 0.11 (0.003–0.35), and 0.06 (0.01–0.20), respectively.

### Changes in clinical characteristics induced by treatment

After examining the variations in standardized differences in the baseline clinical characteristics depending on the utilized weighting model, we adopted the ATE weighting model for further analysis, followed by trimming using a PS value, because this model showed the smallest median value of the standardized differences. The changes in the clinical characteristics after SGLT-2i treatment compared with GLP-1Ra treatment by analysis of the generalized linear model are shown in Table 6. Significant decreases in DBP, MAP, and BW were observed in SGLT-2i- compared with GLP-1Ra-treated patients.

**Table 6:** The changes of the clinical characteristics after SGLT2i treatment compare to GLP1Ra treatment by the analysis of the generalized linear model.

Truncation/trimming	ATE		ATT		Stabilized ATE	
	Model A	Model B	Model A	Model B	Model A	Model B
ΔSBP	− 5.8 [− 12.6, 1.0]/0.09	− 4.5 [− 10.6, 1.5]/0.14	− 7.1 [− 15.0, 0.9]/0.08	− 5.8 [− 9.8, − 1.7]/0.006	− 6.5 [− 13.0, − 0.0]/0.049	− 4.5 [− 10.6, 1.5]/0.14
ΔDBP	− 3.2 [− 6.9, 0.6]/0.10	− 3.8 [− 6.6, − 1.1]/0.006	− 3.3 [− 8.2, 1.7]/0.20	− 4.2 [− 7.2, − 1.1]/0.008	− 3.2 [− 7.0, 0.6]/0.10	− 3.8 [− 6.6, − 1.1]/0.006
ΔMAP	− 4.0 [− 8.3, 0.2]/0.06	− 4.1 [− 7.2, − 1.0]/0.01	− 4.5 [− 9.9, 0.9]/0.10	− 4.7 [− 7.6, − 1.8]/0.001	− 4.3 [− 8.5, − 0.1]/0.046	− 4.1 [− 7.2, − 1.0]/0.01
ΔBW	− 1.7 [− 2.7, − 0.7]/0.001	− 1.5 [− 2.7, − 0.4]/0.008	− 2.0 [− 3.3, − 0.8]/0.002	− 1.9 [− 0.6, − 0.6]/0.003	− 1.7 [− 2.7, − 0.6]/0.001	− 1.5 [− 2.7, − 0.4]/0.008
ΔHbA_1c_	0.6 [− 3.4, 4.6] (0.1 [− 0.3, 0.4])/0.78	− 1.5 [− 5.8, 2.8] (− 0.14[− 0.53, 0.26])/0.49	0.6 [− 4.2, 5.5] (0.1 [− 0.4, 0.5])/0.79	− 2.0 [− 6.7, 2.6] (− 0.2 [− 0.6, 0.2])/0.39	0.7 [− 3.4, 4.7] (0.1 [− 0.3, 0.4])/0.74	− 1.5 [− 5.8, 2.8] (− 0.1 [− 0.5, 0.3])/0.49
ΔeGFR per year	2.6 [− 0.7, 5.8]/0.12	1.5 [0.05, 2.9]/0.04	3.4 [− 0.1, 2.3]/0.13	1.9 [0.1, 3.6]/0.04	2.6 [− 0.7, 5.9]/0.12	1.5 [0.1, 2.9]/0.04
ΔLNACR	− 0.11 [− 0.23, 0.01]/0.07	− 0.13 [− 0.27, 0.02]/0.08	− 0.08 [− 0.23, 0.06]/0.27	− 0.14 [− 0.32, 0.03]/0.11	− 0.12 [− 0.24, 0.002]/0.054	− 0.13 [− 0.27, 0.02]/0.08

Data present as the difference [95% CI]/*P*-value.

The truncation on 99 percentiles is utilized in model A, and the trimming by 0.05 ≦ PS ≦ 0.95 is utilized in model B.

*Δ* change, *BW* body weight, *CI* confidence interval, *DBP* diastolic blood pressure, *eGFR* estimated glomerular filtration rate, *HbA*_*1c*_ glycated hemoglobin A_1c_, *LnACR* logarithmic value of urine albumin-to-creatinine ratio, *MAP* mean arterial pressure, *SBP* systolic blood pressure.

## Discussion

We performed two retrospective cohort surveys to assess the effects of two new classes of antidiabetic drugs, SGLT-2i and GLP-1Ra, on renal function. In our previous analysis, we found that SGLT-2i has superior renoprotective effects compared to GLP-1Ra and that BP reduction caused by treatment with SGLT-2i contributed to this effect^24,26^. To compare the efficacy of the two classes of drugs in lowering BP, we selected patients with poorly controlled baseline BP levels. SGLT-2i treatment showed a better BP control rate than GLP-1Ra treatment, which underlies the renoprotective effects of SGLT-2i treatment.

Although randomized controlled studies provide high-quality evidence, retrospective cohort studies have serious limitations due to inadequate data on confounding factors. PS is the probability that a case is included in the treated group and is calculated using background characteristics that are considered confounding factors. PS methods have been used to control confounding factors in clinical studies^27^. Statistical methods using PS, such as PS weighting and matching, may be useful in estimating treatment effects^28^. Among these, the PS-matching method reduces the effects of confounding factors by selecting only participants with close PS and has been used in some real-world studies, such as CVD-REAL (Comparative Effectiveness of Cardiovascular Outcomes in New Users of SGLT-2 Inhibitors)^29^, EMPRISE (Empagliflozin Comparative Effectiveness and Safety)^30^, and the J-CKD database^31^. These studies have evaluated the cardiovascular and renal outcomes of SGLT-2i treatment.

PS weighting is a PS-based statistical method. In this survey, we selected participants who did not achieve their target BP at baseline. Consequently, the sample size of both the treatment groups decreased. We obtained extreme PS between the two treatment groups in the present models (the value of c-statistics was 0.904); therefore, PS matching may result in a substantial loss of sample size. Consequently, we selected the PS-weighting method for further analysis.

Different weighting methods can be used depending on the primary treatment effect of interest (ATE, stabilized ATE, or ATT). The formula for the weight calculation using PS is shown in Supplementary Fig. S2. When the value of PS approaches zero or one, the weight value is extremely high, resulting in biased estimates and excessive variance.

Lee et al. reported that trimming large weights could improve the performance of PS weighting^32^. Symmetric trimming, which excluded subjects with PS values ranging from 0.05 to 0.95, was reported by Richard^33^. The truncation of the weight for values > 99 percentiles was reported by Cole and Hernan^34^. Currently, there is no consensus on the best method for choosing the correct PS-weighting, trimming, or truncation method. Six statistical models were used in the survey.

To avoid selecting models that would generate the most favorable results, we assessed the integrated OR as the primary outcome, calculated using a meta-analysis of data resulting from the six models applied. We concluded that SGLT-2i treatment offers superior control of BP in T2DM patients compared to GLP-1Ra treatment. Some concerns regarding this analysis must be mentioned. The ORs of the individual models were calculated using a generalized linear model analysis. The integrated OR was calculated by combining the above-mentioned ORs in the meta-analysis and using the weighted number of events. Therefore, the OR values, including the lower and upper 95% CIs, were not completely matched. Further analysis and discussion are warranted to determine the best method for assessing the OR and choosing the optimal PS weighting model. It may be difficult to define an appropriate method for PS weighting because the standardized differences had a large variance in each model utilized, as illustrated in Supplementary Fig. S4. Further analysis is needed to determine which model is appropriate for this study, using data from clinical practice.

A meta-analysis showed the predominance in composite renal outcome of SGLT-2i and GLP-1Ra treatment compared with placebo (45% HR, 0.55; 95% CI 0.48–0.64; *P* < 0.0001)^35^; (17% HR, 0.83; 95% CI 0.78–0.89; *P* = 0.098)^36^, respectively. A network meta-analysis concluded that SGLT-2i treatment was superior to GLP-1Ra treatment^37,38^. The renoprotective mechanism of SGLT-2is may be a result of multiple factors: favorable effects on vascular function by reducing intraglomerular pressure through restoration of tubuloglomerular feedback, improvement of hypoxia in the proximal kidney tubule, and metabolic effects, such as a reduction in BP or BW^39,40^. In turn, the renoprotective effects of GLP-1Ras may be a consequence of increased sodium excretion due to the inhibition of the sodium-hydrogen exchanger isoform 3^41^ or anti-inflammatory and antioxidant effects^42,43^. In our previous studies, we found that SGLT-2i treatment was associated with a larger decrease in BP than GLP-1Ra treatment, and that this effect was strongly correlated with their renoprotective effects^25^. Regarding the secondary outcome, we selected the ATE weighting model associated with PS-based trimming to generate minimal standardized differences. Using this model for further analysis, we found that SGLT-2i treatment resulted in a greater decrease in BP and BW, and higher eGFR values than GLP-1Ra treatment. These results are similar to those of our previous study^25^. There was no significant difference in the decrease in LnACR between the two groups. However, a larger sample size may show that SGLT-2i treatment results in a greater decrease in LnACR than GLP-1Ra treatment. Accordingly, the renoprotective effect of SGLT-2i in T2DM patients with poorly controlled BP was superior to that of GLP-1Ra.

Tsapas et al. reported on the comparative efficacy of hypoglycemic drugs on BW in patients with T2DM using network meta-analysis^44^. SGLT-2is and GLP-1Ras treatments showed a significant decrease in BW from − 0.8 kg (dulaglutide) to − 3.8 kg (semaglutide by weekly subcutaneous injection) compared to placebo. There seemed to be no significant differences in the decrease in BW between SGLT-2is and GLP-1Ras. However, the decrease in BW with dulaglutide; − 0.80 (95% CI  − 1.41, − 0.19) tended to be smaller than that of other GLP-1Ras and SGLT-2is. In this study, dulaglutide was administered to 33.1% of patients treated with GLP-1Ra, which may have influenced a significantly larger decrease in BW by SGLT2i than GLP-1Ras, that can induce proper BP management.

The prevalence of postprandial hypotension (a decrease in SBP by at least 20 mmHg within 2 h after meals) is high in patients with T2DM^45^. The use of GLP-1Ras, especially short-acting GLP-1Ras (exenatide and lixisenatide), has the potential to slow gastric emptying and prevent this phenomenon. The breakdown of GLP-1Ras in this study was 70 (43.8%) liraglutide, 53 (33.1%) dulaglutide, 5 (3.1%) exenatide, 2 (1.3%) lixisenatide, and 30 (18.8%) patients changed the GLP-1Ra type during the observation period. The unadjusted change in MAP after treatment was − 4.7 ± 11.4 mmHg in 123 patients treated with long-acting GLP-1Ras and − 5.2 ± 4.0 mmHg in 7 patients treated with short-acting GLP-1Ras. However, no significant difference was observed because of the small sample size. Future studies are needed to clarify the effect of hypoglycemic drug-induced postprandial hypotension on BP management.

### Study limitations

The standardized differences in clinical characteristics were below 0.20, with some exceptions, which were higher than 1.0, even in this model. In general, a standardized difference of < 0.10 is associated with a meaningful imbalance between the two groups^46^. Austin reported standardized differences exceeding 0.20 are expected, even when the PS-matched model is correctly specified^47^. The model used in the current study (ATE weighting associated with PS-based trimming) could decrease the imbalance between clinical characteristics at baseline. However, bias remains concerning the confounding factors that were not included in the study. This is a major limitation of this study.

In addition to the above-mentioned points, our comparative study has some limitations. Our surveys were small in size, retrospective, and observational, were not performed simultaneously, and did not have the same inclusion criteria. The subjects of the SGLT-2i survey were patients with T2DM and CKD, whereas those of the GLP-1Ra survey were only patients with T2DM, thus suggesting the possibility of selection bias. In clinical practice, combining both drugs are becoming a basic strategy for managing T2DM, and only patients with underlying contraindications to one of them receive the other. In this study, we compared patients receiving only one of these treatments. Therefore, our results are not applicable to patients treated with combination therapy. As only 265 GLP-1Ra-treated patients were included in the comparative analysis, selection bias appears to be a major issue in our survey.

Furthermore, our survey included only data from subjects who received continuous treatment. No data on adverse events that emerged during treatment were collected. Adverse events and quality of life (QOL) are important outcomes of treatment. SGLT-2i drugs are administered orally, whereas GLP-1Ra drugs are administered by injection (oral semaglutide was not available in Japan at the time of the survey), which may have resulted in a lower QOL or adherence to GLP-1Ra treatment. These factors may have further influenced our results.

Given that participants had a high cardiovascular risk (diabetes with high HbA_1c_ levels, uncontrolled hypertension, and most predominantly chronic kidney disease), the rate of use of metformin, RASis, or statins could be low. These low rates may be considered as “insufficient management for high-risk patients.” This is one of the most important and serious limitations of our study; however, at the same time, these situations reflect the actual clinical situation in Japan. Nagasu et al. reported the renoprotective effect of SGLT-2i treatment using the Japan Chronic Kidney Disease Database (J-CKD-DB), a nationwide multicenter CKD registry^31^. In the J-CKD-DB, the rates of metformin, RASi, diuretic, and statin use in the SGLT-2i group were 56.7%, 46.2%, 10.8%, and 47.7%, respectively. These rates were not markedly different from those in our study, although the participants included in the studies and the types of medical institutions were not necessarily the same. Many reasons for the low rates of these medications are suspected, for example, fear of adverse effects, adherence of patients to treatment, risk of polypharmacy, or performance of patient-centered therapy. The conclusion may differ when more powerful concomitant medications are administered to patients at a high risk of renal events. A previous study showed that concomitant insulin treatment with SGLT-2i was significantly associated with a high risk of renal composite outcomes using the PS matching method^48^. The rate of sulfonylurea use in the insulin (+) group was significantly lower than that in the insulin (−) group even after PS matching (17.5% and 44.5%, respectively; *P* < 0.01). Although the use of sulfonylurea was not identified as an independent risk factor for renal composite outcomes by logistic regression analysis in our previous study of SGLT-2i^20^, further study is needed to reveal the relationship between the use of sulfonylurea and renal composite outcomes.

Furthermore, statistical methods using PS may be useful for estimating treatment effects; however, there are many concerns that need to be addressed, for example, statistical multiplicity or a study protocol that has not determined the statistical method in advance. Only a perfectly randomized control study can eliminate the concern for confounding factors, and in fact, our clinical retrospective observational study has many limitations and concerns. However, we believe that high-level statistical analysis has contributed to reducing the effects of confounding factors and that the results of our study will be of some help for future research.

Taken together, our results demonstrate that in T2DM patients with poorly controlled BP, SGLT-2i treatment significantly improved BP management (the integrated OR and 95% CI was 2.09 [1.80, 2.43]) and increased BW loss and eGFR compared to GLP-1Ra treatment (*P* = 0.01, and 0.04, respectively). The efficacy in achieving the target BP rate and the characteristics of antidiabetic drugs should be considered to accomplish renoprotective effects in clinical practice.

## Methods

### Study patients and data collection

This study is a subanalysis of our previous study, and the methods used in this study are described in our previous report^25^. Briefly, the Kanagawa Physicians Association conducted two retrospective surveys that included patients with T2DM receiving SGLT-2i or GLP-1Ra therapy to investigate their influence on renal function. The studies included patients who visited the clinics of members of the association between October and December 2018 and between July and October 2020 for the SGLT-2i and GLP-1Ra surveys, respectively. Both surveys had the following inclusion criteria: patients with T2DM who were (a) treated with each drug for more than one year, (b) aged over 20 years, and (c) diagnosed as having CKD, as defined by the K/DOQI clinical practice guidelines (only for SGLT-2i retrospective study)^49^. The following patients were excluded: (a) undergoing chronic dialysis, (b) type 1 DM, (c) severe liver dysfunction (e.g., liver cirrhosis or severe hepatitis), (d) stage IV malignancy, (e) pregnancy, (f) poor adherence to each drug (suggested by irregular use), and (g) intending to opt out during the study. Applying these criteria, 34 patients from the SGLT-2i survey and 33 from the GLP-1Ra survey were excluded.

The following parameters were recorded at the time of initiation of each treatment and at the time of the survey: age, sex, BW, BP (SBP, DBP, MAP), serum creatinine level, glycated hemoglobin A_1c_ (HbA_1c_), urinary ACR [mg/g Cr], and qualitative proteinuria. The estimated glomerular filtration rate (eGFR) was calculated using the following formula: eGFR (mL/min/1.73 m^2^) = 194 × age^−0.287^ × serum creatinine^−1.094^ × (0.739 for women)^50^. The formula reported by Sumida et al.^51^ was used to convert qualitative proteinuria values to albuminuria values. Documentation of patients’ medical records was extracted by medical doctors and anonymized patient forms were used.

BP measurements at the office were performed at each institution using validated cuff oscillometric devices. According to the Japanese Society of Hypertension Guidelines for the Management of Hypertension (JSH 2019) guidelines^52^, BP at the office was measured in a quiet environment after resting for a few minutes in a seated position on a chair with the legs uncrossed. Attended or unattended was not specified. Two consecutive measurements were taken 1–2 min apart, and the average of the two measurements was defined as the BP at the office.

A total of 140 patients in the SGLT-2i survey and 29 patients in the GLP-1Ra survey were excluded because of missing ACR values at any point during data collection. Patients concomitantly treated with both drugs were excluded. Thus, 541 and 265 patients were included in the SGLT-2i survey (SGLT-2i group) and GLP-1Ra survey (GLP-1Ra group), respectively. Patients with poorly controlled BP (> 130/80 mmHg; determined in the clinical setting before the initiation of therapy) were further selected. In total, 384 and 160 patients were included in the SGLT-2i and GLP-1Ra groups, respectively.

### Outcomes

In accordance with JSH 2019, the target BP for patients with T2DM is < 130/80 mmHg^52^. The primary endpoint of this study was the achievement rate of target BP.

### Statistical analysis

When different retrospective surveys are utilized to appropriately analyze the differences in the outcomes of the two treatments, adjustment for confounding factors is needed. PS analysis is useful for balancing the confounding factors between the two groups. The PS values for SGLT-2i-treated patients were calculated using a logistic regression model to estimate the probability of treatment efficacy when considering the following variables: age, sex, BW, HbA_1c_, SBP, DBP, eGFR, and the logarithmic value of LnACR at baseline, as well as the concomitant use of antihypertensive drugs, other glucose-lowering drugs, and statins. All of these factors are considered common confounding factors when assessing the efficacy of antihypertensive treatment.

In this study, the IPW method was used for comparative analysis. Several methods of weighting and trimming values can be utilized when the IPW method is applied. As no method is currently considered the best, we utilized three weighting methods depending on the primary treatment effect of interest: ATE weighting, ATT weighting, or stabilized ATE weighting. Supplementary Fig. S2 illustrates the calculation method for each weight using the PS. Two methods were further used for the adjustment of IPW: “weight truncation” that consisted of the exclusion of weights larger than 99 percentiles (model A), or “weight trimming” that consisted of excluding extreme PS values and including only patients with PS ranging from 0.05 to 0.95 (model B). Accordingly, six models were used in the IPW method. Regarding the primary outcome, ORs were calculated using the data obtained from the six models, and a meta-analysis of the data was conducted to calculate the integrated OR using EZR version 1.55 (Saitama Medical Center, Jichi Medical University, Saitama, Japan).

Regarding other comparative analyses, the model leading to the smallest standardized differences between the clinical characteristics at baseline was selected, and a generalized linear model analysis was performed for the comparison between groups.

An unpaired *t* test was used to analyze differences in the clinical and laboratory pathological profiles between the two groups. The Mann–Whitney rank-sum test was used for continuous variables, and the chi-square test was used for categorical data in the cohort model before applying IPW. After applying IPW, a generalized linear model was used for the comparative analysis.

The results are presented as mean ± standard deviation, mean (lower and upper 95% CI), or median with interquartile range (IQR) for continuous data and as percentages for categorical data. Statistical significance was set at *P* < 0.05. All statistical analyses, except for the calculation of the integrated OR for the primary outcome, were performed using the IBM SPSS Statistics software (version 28.0; IBM Inc., Armonk, NY, USA).

This retrospective study was conducted in accordance with the principles of the Declaration of Helsinki and was approved by the Special Ethics Committee of the Kanagawa Medical Association, Japan (approval Krec202005 on March 23, 2020, for the GLP-1Ra survey and this comparison survey; approval Krec304401 on March 6, 2018, for the SGLT-2i survey). The requirement for informed consent was waived by the special ethics committee of the Kanagawa Medical Association, Japan, owing to the retrospective and observational nature of the study.

## Supplementary Information

Supplementary Figures.

## Data Availability

Data are available from the Kanagawa Physicians Association Data Access/Ethics Committee for investigators and are bound by confidentiality agreements. Contact details: Kazuo Kobayashi MD, Kanagawa Physicians Association, 3-1Fujimicho Naka-ku, Yokohama City, Kanagawa Prefecture, Japan E-mail: k-taishi@xc4.so-net.ne.jp.
