# Supplementary material for: Comparison of the blood pressure management between sodium-glucose cotransporter 2 inhibitors and glucagon-like peptide 1 receptor agonists
Source: Sci Rep. 2022 Sep 27;12:16106. doi: 10.1038/s41598-022-20313-5 (PMC9515152; doi:10.1038/s41598-022-20313-5)
Supplement: Supplementary file 1 — Supplementary Figures. [file 41598_2022_20313_MOESM1_ESM.pdf]

Supplementary Figures

Supplementary Figure S1 The schema of the subjects included in this survey

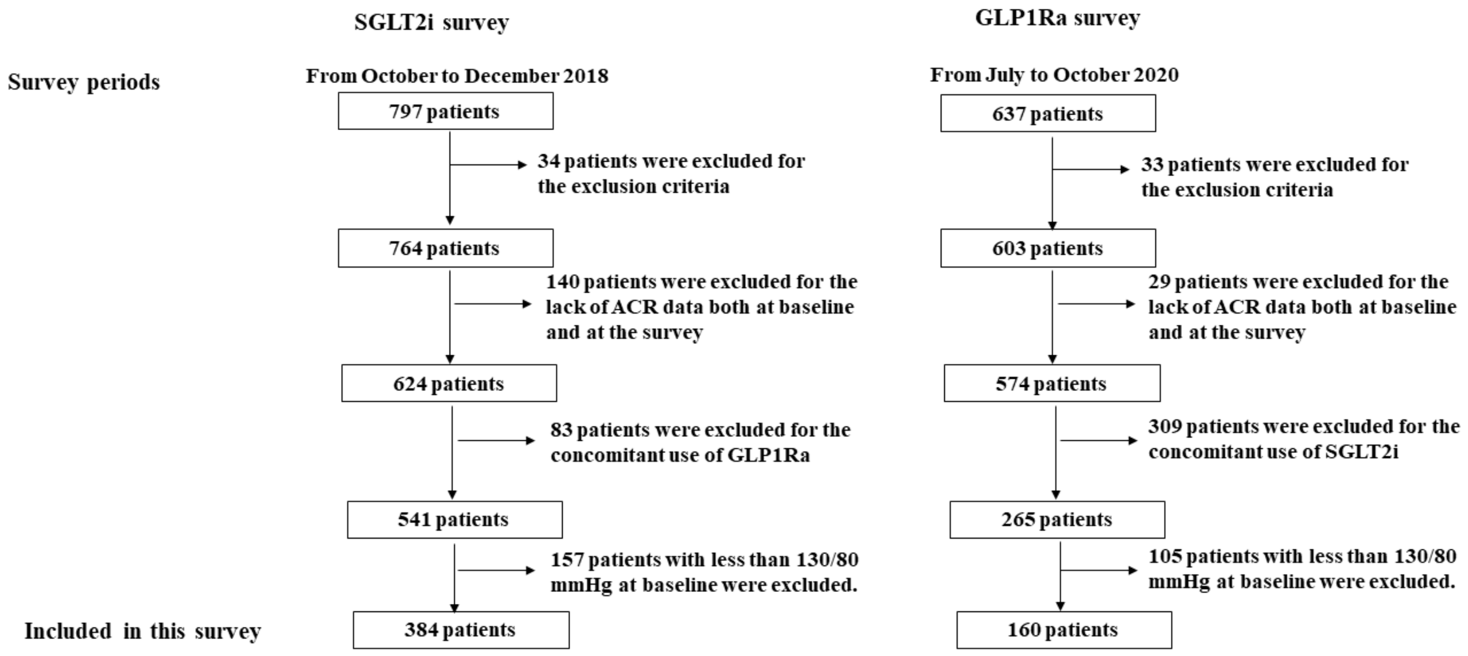

Abbreviation; GLP1Ra; glucagon-like peptide 1 receptor agonist; SGLT2i, sodium-glucose co-transporter inhibitor

Supplementary Figure S2 The calculation of the weight using propensity score

	The SGLT2i treated patients	The GLP1Ra-treated patients
ATE weight	1/PS	1 / (1 - PS)
ATT weight	1	PS / (1 - PS)
Stabilized ATE weight	p / PS	(1 - p) / (1 – PS)

The p value is the ratio of the SGLT2i-treated patients in total patients.

Abbreviation; ATE, average treatment effect; ATT, average treatment effect on the treated; GLP1Ra; glucagon-like peptide 1 receptor agonist; PS, propensity score; SGLT2i; sodium-glucose co-transporter inhibitor

Supplementary Figure S3 The distribution of the propensity sore of SGLT2i-treated and GLP1Ra-treated patients

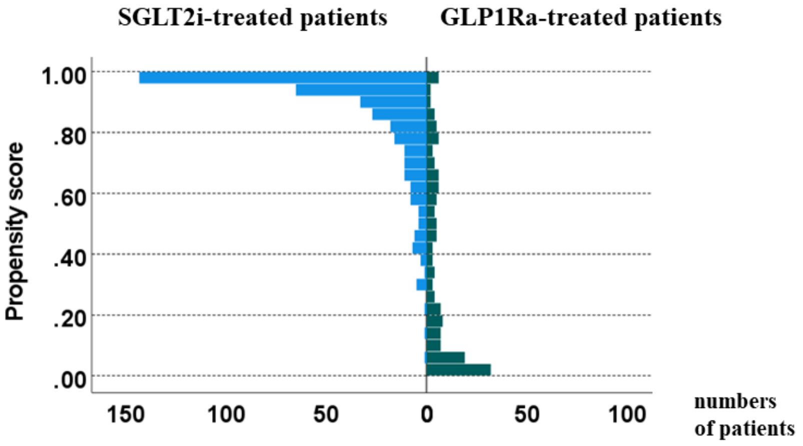

Abbreviation; GLP1Ra, glucagon-like-1 receptor agonist; SGLT2i, sodium glucose cotransporter inhibitor

Supplementary Figure S4 The standardized difference of the clinical characteristics at baseline depending on six models for weighting

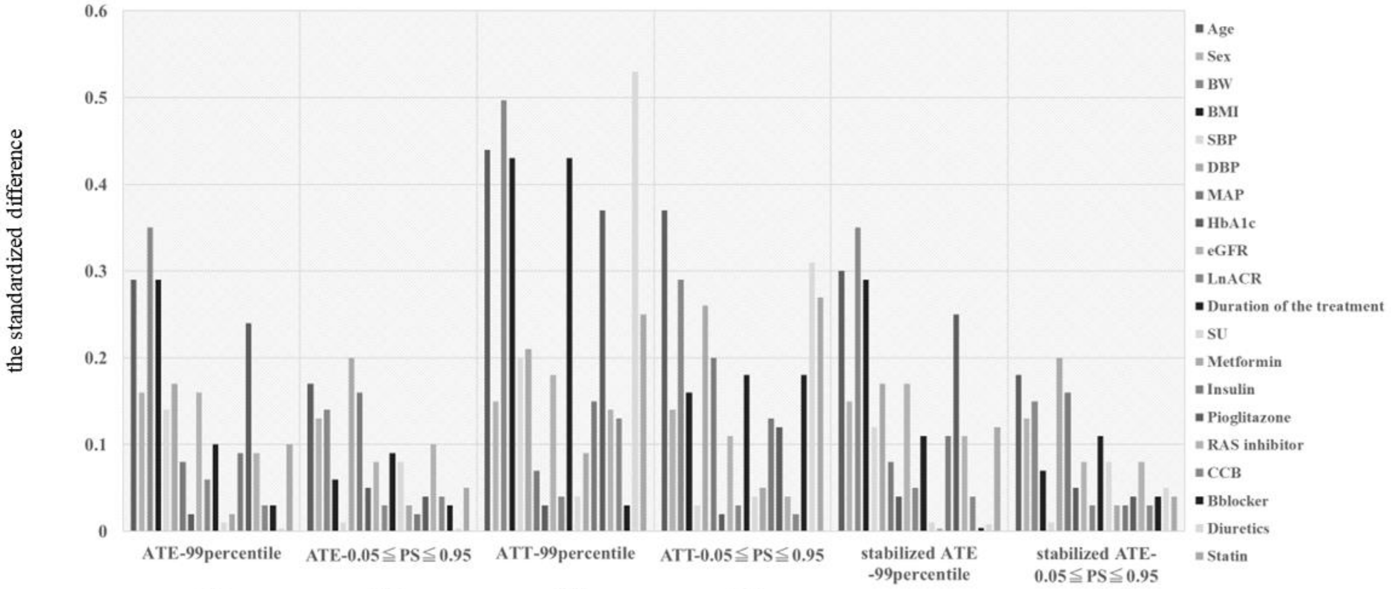

The median (range) of the standardized difference	**0.10 (0.003-0.35)**	**0.06 (0.003-0.20)**	**0.17 (0.03-0.5)**	**0.14 (0.02-0.37)**	**0.11 (0.003-0.35)**	**0.06 (0.01-0.20)**

The truncation on 99 percentiles is utilized in model A, and) the trimming by 0.05≦PS≦0.95 is utilized in model B.

Abbreviation; BMI, body mass index; BW, body wight; CCB, calcium channel blocker; DBP, diastolic blood pressure; eGFR, estimated glomerular filtration; HbA_{1c}, glycated hemoglobin A_{1c}; IPW, inverse probability weighting; LnACR, logarithmic value of urine albumin-to-creatinine ratio; ; MAP, mean arterial pressure; PS, propensity score; RAS, renin aldosterone system; SBP, systolic blood pressure; SGLT2i, sodium glucose cotransporter inhibitor; SU, sulphonyl urea
